# Engineering extracellular vesicles for targeted therapeutics in cardiovascular disease

**DOI:** 10.3389/fcvm.2024.1503830

**Published:** 2024-12-19

**Authors:** Enze Fu, Kai Pan, Zongjin Li

**Affiliations:** ^1^ School of Medicine, Nankai University, Tianjin, China; ^2^ Institute of Ophthalmology, Nankai University, Tianjin, China; ^3^ Henan Key Laboratory of Cardiac Remodeling and Transplantation, Seventh People's Hospital, Zhengzhou, China; ^4^ National Key Laboratory of Kidney Diseases, Chinese PLA General Hospital, Beijing, China

**Keywords:** extracellular vesicles (EVs), cardiovascular diseases (CVDs), engineering, multiomics analysis, targeted therapeutics

## Abstract

Extracellular vesicles (EVs) are nanosized particles secreted by cells that play crucial roles in intercellular communication, especially in the context of cardiovascular diseases (CVDs). These vesicles carry complex cargo, including proteins, lipids, and nucleic acids, that reflects the physiological or pathological state of their cells of origin. Multiomics analysis of cell-derived EVs has provided valuable insights into the molecular mechanisms underlying CVDs by identifying specific proteins and EV-bound targets involved in disease progression. Recent studies have demonstrated that engineered EVs, which are designed to carry specific therapeutic molecules or modified to enhance their targeting capabilities, hold promise for treating CVDs. Analysis of the EV proteome has been instrumental in identifying key proteins that can be targeted or modulated within these engineered vesicles. For example, proteins involved in inflammation, thrombosis, and cardiac remodeling have been identified as potential therapeutic targets. Furthermore, the engineering of EVs to increase their delivery to specific tissues, such as the myocardium, or to modulate their immunogenicity and therapeutic efficacy is an emerging area of research. By leveraging the insights gained from multiomics analyses, researchers are developing EV-based therapies that can selectively target pathological processes in CVDs, offering a novel and potentially more effective treatment strategy. This review integrates the core findings from EV multiomics analysis in the context of CVDs and highlights the potential of engineered EVs in therapeutic applications.

## 1 Introduction

Cardiovascular diseases (CVDs) remains a leading cause of mortality and morbidity worldwide, posing significant challenges to global health. Despite advances in medical technology and therapeutics, the burden of CVDs continues to grow, driven by factors such as aging populations, lifestyle changes, and the increasing prevalence of risk factors such as hypertension, diabetes, and obesity (1). Although effective to some extent, traditional therapeutic approaches often fail to address the complex and multifactorial nature of CVDs fully. This has led to a growing interest in innovative treatment strategies that can more precisely target the underlying pathophysiological mechanisms of the disease (2). One promising area of research focuses on the use of EVs as novel therapeutic agents because of their natural role in intercellular communication and their ability to carry and deliver a diverse array of biomolecules (3).

EVs are nanosized particles secreted by virtually all cell types and are increasingly recognized for their critical role in mediating intercellular communication. EVs include a variety of subtypes, including exosomes, microvesicles, and apoptotic bodies, each distinguished by their size, biogenesis, and molecular composition (4). These vesicles carry a complex cargo of proteins, lipids, nucleic acids, and other biomolecules, which reflect the physiological or pathological state of their cells of origin. Exosomes (30–150 nm) carry proteins, RNA, and lipids and are formed through the inward budding of endosomes, with release via the fusion of multivesicular bodies with the plasma membrane. Microvesicles (100–1,000 nm), which are shed directly from the plasma membrane, contain cytoskeletal proteins, heat shock proteins, integrins, and posttranslationally modified proteins such as glycosylated and phosphorylated proteins. Apoptotic bodies (1–5 µm), which are released during cell death, contain complete organelles, chromatin, and a small amount of glycosylated proteins (5). In the context of CVDs, EVs influence various aspects of disease progression, including inflammation, thrombosis, and cardiac remodeling, by transferring bioactive molecules between cells (6). This capacity to modulate cellular behavior makes EVs attractive candidates for therapeutic applications, particularly in the targeted treatment of CVDs.

The potential of EVs as therapeutic agents is further amplified by advancements in multiomics technologies, which have allowed for a comprehensive analysis of the molecular cargo within these vesicles. Multiomics analyses, including proteomics, genomics, transcriptomics, and metabolomics, have provided valuable insights into the molecular mechanisms underlying CVDs by identifying specific proteins and EV-bound targets involved in disease progression (7). These findings not only deepen our understanding of the pathophysiology of CVDs but also open new avenues for the development of engineered EVs designed to carry specific therapeutic molecules or to enhance their targeting capabilities. Moreover, the integration of these omics-driven insights with innovative engineering techniques has enabled the creation of EVs with enhanced therapeutic properties, including improved delivery efficiency, tissue specificity, and reduced immunogenicity (8). This review aims to integrate the core findings from EV multiomics analyses in the context of CVDs and to highlight the emerging potential of engineered EVs in therapeutic applications. By exploring the intersection of EV biology, multiomics research, and bioengineering, this review seeks to provide a comprehensive overview of how engineered EVs could revolutionize the treatment of CVDs, offering novel strategies to address its complex and multifaceted nature.

## 2 Composition and function of EVs

### 2.1 Molecular cargo of EVs

EVs are intricate and biologically active particles secreted by cells that carry diverse cargos, including proteins, lipids, nucleic acids, and metabolites. The composition of these vesicles is closely linked to the cell of origin and its physiological or pathological state (9). Proteins within EVs often consist of enzymes, signaling molecules, and structural components that facilitate their role in intercellular communication and the modulation of recipient cell functions. Lipids, which form the vesicle membrane, not only maintain structural stability but also play a critical role in the interaction of EVs with target cells, influencing their uptake and cargo delivery. EVs are characterized by a diverse molecular cargo that reflects their cellular origin and biological functions. Key protein markers of EVs include tetraspanins (CD9, CD63, and CD81), play critical roles in EVs structure and function. These proteins are involved in the organization of membrane microdomains, facilitating the sorting of specific cargo and mediating interactions with recipient cells. CD63, for example, is associated with late endosomal compartments and is crucial in EVs formation, while CD9 and CD81 are linked to membrane fusion events during EVs release or uptake (5, 10, 11). Endosomal Sorting Complex Required for Transport (ESCRT)-associated proteins (TSG101 and Alix), which are crucial for EV biogenesis and serve as evidence of their endosomal origin. TSG101, a component of the ESCRT-I complex, is essential for EVs biogenesis, specifically in the sorting of ubiquitinated proteins into intraluminal vesicles (ILVs) within multivesicular bodies (MVBs). Alix, another key ESCRT-associated protein, interacts with ESCRT-III components and facilitates the budding of ILVs into MVBs (12, 13). Heat shock proteins, such as HSP70 and HSP90, contribute to EV stability and immune-modulatory functions (14, 15). Lipids, including cholesterol, sphingolipids, ceramides, and phosphatidylserine, further distinguish EV membranes, enhancing their stability and recipient cell interactions. Additionally, RNA-binding proteins like hnRNPA2B1 and AGO2 mediate selective RNA packaging, underscoring the regulatory potential of EV cargo (16, 17). Nucleic acids such as mRNAs, miRNAs, and other noncoding RNAs are key functional elements within EVs, enabling them to modulate gene expression and cellular responses in recipient cells (18). This molecular versatility positions EVs as crucial mediators of various biological processes, particularly in the context of cardiovascular health and disease.

### 2.2 Functional role of EVs in cardiovascular disease

EVs uptake is a complex, multifaceted process critical for their role in cell-to-cell communication and therapeutic applications. EVs interact with recipient cells through various pathways, often dictated by the molecular composition of the EVs membrane, such as tetraspanins, integrins, and glycoproteins, as well as the surface receptors of the target cell (19). Uptake mechanisms include clathrin-mediated endocytosis, where receptor-ligand interactions trigger vesicle internalization through clathrin-coated pits, and caveolin-mediated pathways, which utilize lipid raft domains (20–22). Macropinocytosis, an actin-driven process, allows non-specific engulfment of EVs, while phagocytosis is predominantly used for larger EVs and involves their recognition by phagocytic receptors (23, 24). Direct fusion of EV membranes with the plasma membrane or endosomal membranes following endocytosis provides an alternative route for cargo delivery (25, 26). The uptake route may vary depending on the molecular composition of both the EVs and the target cell, suggesting a heterogeneous and complex entry process.

Cardiovascular pathologies including a wide range of conditions, including atherosclerosis, myocardial infarction, hypertension, and heart failure, which collectively represent the leading causes of morbidity and mortality globally. These diseases are characterized by multifaceted processes involving chronic inflammation, oxidative stress, and endothelial dysfunction, which disrupt vascular homeostasis and promote plaque formation, thrombosis, and vascular remodeling (27, 28). Atherosclerosis, driven by lipid accumulation and immune cell infiltration, serves as a precursor to many acute events such as myocardial infarction, where ischemia and subsequent tissue damage result in cardiomyocyte death and fibrosis (29). Hypertension exacerbates vascular injury through increased shear stress and endothelial cell activation, further contributing to target organ damage (30). Heart failure, often a consequence of ischemic injury or chronic pressure overload, involves maladaptive remodeling, impaired cardiac output, and activation of neurohormonal pathways (31). These interconnected processes highlight the complexity of cardiovascular disease, necessitating novel therapeutic strategies to address its underlying mechanisms.

In CVDs, EVs have emerged as promising therapeutic agents capable of supporting and enhancing cardiovascular health through various mechanisms. One of the primary therapeutic roles of EVs in CVDs is their ability to facilitate tissue repair and regeneration, particularly following MI (32). EVs derived from stem cells, such as mesenchymal stem cells (MSCs) or cardiac progenitor cells (CPCs), have been shown to carry regenerative factors, including growth factors, antiapoptotic proteins, and proangiogenic miRNAs (33, 34). These EVs can be taken up by cardiac cells, where they promote cell survival, reduce apoptosis, and stimulate angiogenesis, thereby assisting in the repair of damaged myocardial tissue. For example, EVs enriched with miR-21 have been demonstrated to reduce fibrosis and enhance the regenerative capacity of the heart by targeting pathways involved in apoptosis and inflammation (35). Additionally, EVs carrying vascular endothelial growth factor (VEGF) can promote the formation of new blood vessels, improving the blood supply to ischemic areas and facilitating tissue recovery (36, 37). These therapeutic actions underscore the potential of EV-based therapies to mitigate the damage caused by acute cardiovascular events and support long-term recovery.

Another significant role of EVs in CVDs is their capacity to modulate immune responses and reduce inflammation, which is a critical factor in the progression of many cardiovascular conditions. EVs derived from certain cell types, such as regulatory T cells (Tregs) or MSCs, carry anti-inflammatory molecules and immunomodulatory miRNAs that can suppress excessive immune activation and promote a more balanced immune response (38). By delivering these therapeutic molecules to immune cells, EVs can reduce chronic inflammation, which is a key driver of atherosclerosis and other CVDs. For example, EVs carrying miR-146a have been shown to downregulate proinflammatory cytokines and inhibit the activation of NF-*κ*B, a major inflammatory signaling pathway, thereby reducing the inflammatory burden on the cardiovascular system (39). Moreover, a study demonstrated that engineered EVs derived from M2 macrophages (M2 Exo), loaded with hexyl 5-aminolevulinate hydrochloride (HAL) have shown inflammation-tropism, anti-inflammatory effects, and potential for both therapy and imaging in atherosclerosis through targeted cytokine release and HAL metabolism (40). EV-derived circ_0001785 has been identified as a novel biomarker in atherosclerosis and has been shown to reduce endothelial cell injury and delay plaque formation through the miR-513a-5p/TGFBR3 ceRNA network mechanism, providing a potential EV-based therapeutic strategy for atherogenesis (41).

**Figure 1:**
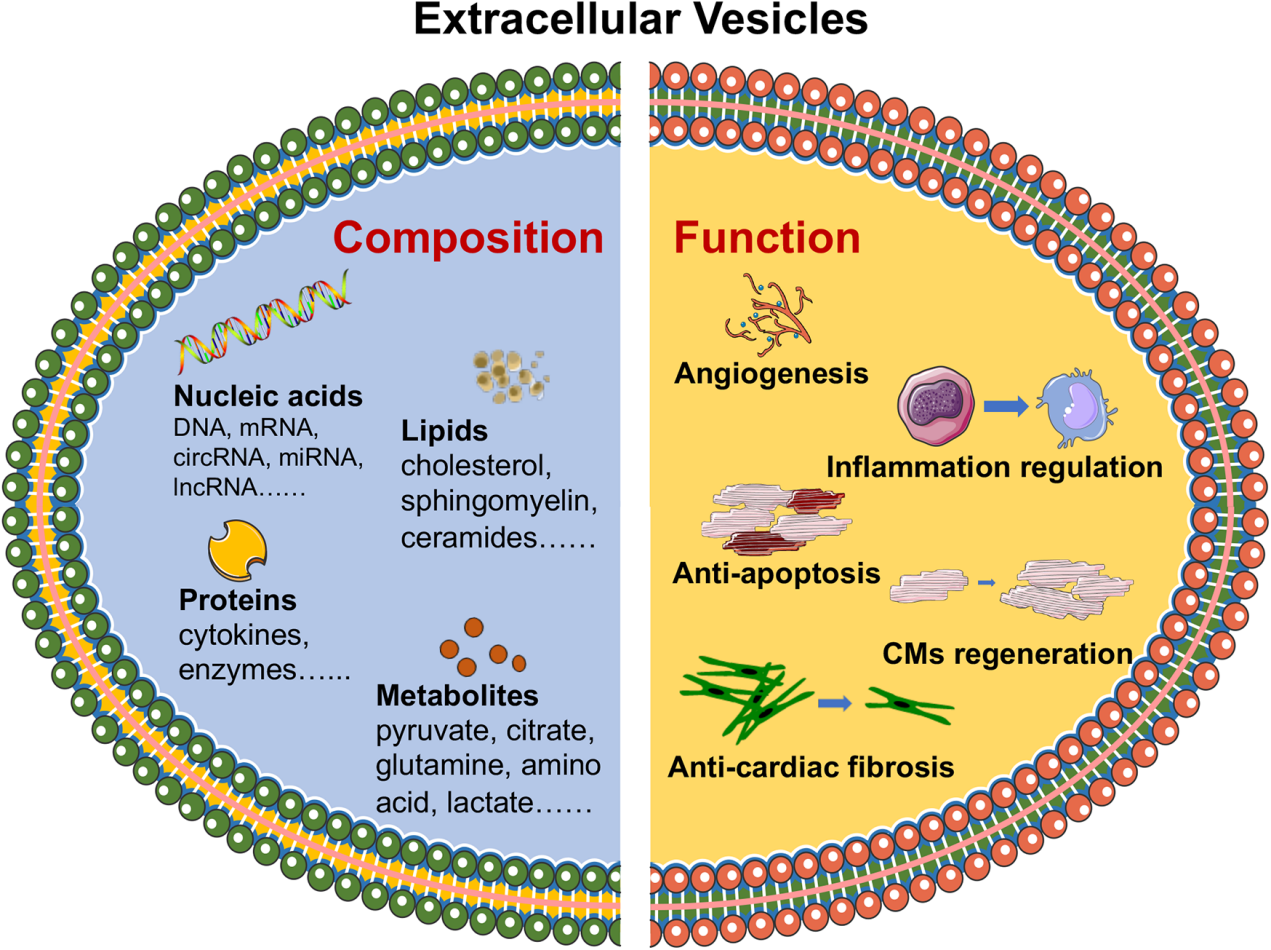
Composition and functional roles of EVs in CVDs. EVs, including proteins, lipids, nucleic acids and metabolites, have been identified as carriers of essential biomolecules that influence key biological processes, such as angiogenesis, inflammation regulation, cardiomyocyte survival and regeneration, and tissue repair. The figure was partly generated via Servier Medical Art, provided by Servier, licensed under a CC BY 3.0.

Extracellular vesicle-packaged noncoding RNAs (ncRNAs) have shown potential as biomarkers and therapeutic targets in hypertension, playing critical roles in vascular remodeling and offering new avenues for the diagnosis and treatment of hypertension-related pathologies. Urinary EV-derived miR-146a has been identified as a potential biomarker for early renal injury in hypertension, with low expression levels strongly associated with albuminuria and capable of discriminating the presence of urinary albumin excretion (42). Mesenchymal stem cell-derived EVs alleviate hypoxic pulmonary hypertension by reducing pulmonary vascular remodeling, right ventricular hypertrophy, and pulmonary artery pressure through the inhibition of the Hsp90aa1/ERK/pERK pathway and the suppression of PASMC proliferation, migration, and resistance to apoptosis (43). Moreover, EVs are involved in processes such as thrombosis and heart remodeling. For example, EVs can carry factors that promote angiogenesis and reduce thrombosis risk, thereby improving vascular function and preventing adverse cardiovascular events (44). In heart remodeling, EVs can deliver miRNAs that modulate fibrotic responses and promote the regeneration of healthy cardiac tissue (45). Through these mechanisms, EVs represent a novel and promising strategy for the treatment of CVDs, with the potential to improve patient outcomes by addressing both the underlying causes and the consequences of cardiovascular pathology (Figure 1).

## 3 Comprehensive multiomics profiling of cell-derived EVs in cardiovascular disease

### 3.1 Integrative multiomics approaches for EVs characterization

Multiomics approaches have revolutionized the study of EVs by providing a comprehensive and integrative view of their molecular cargo. These approaches combine various omics technologies—such as proteomics, genomics, transcriptomics, and metabolomics—to analyze the full spectrum of biomolecules within EVs (7). Proteome profiling revealed that EVs carry selectively enriched protein cargo associated with biological functions such as angiogenesis and inflammation regulation, notably excluding nuclear proteins. This selective cargo is crucial for intercellular communication and tissue repair, particularly in MSCs (46, 47). Using an unbiased proteomic approach based on super-SILAC and high-resolution mass spectrometry, researchers identified 1,212 proteins in the proteome of exosomes, among which 22 proteins were universally enriched, including syntenin-1, a potential universal exosome biomarker (48).

Transcriptomics is used to examine the RNA content, identify genetic material that can be transferred between cells, and influence gene expression and cellular behavior. Research by Zhang et al. revealed that EVs derived from MSCs contain therapeutic miRNA components that may provide a nephroprotective effect (49). Furthermore, a study identifies two distinct extracellular RNA (exRNA) profiles released by mast cells, termed high-density (HD) and low-density (LD) exRNA. While both contain mRNA and miRNA, HD exRNA is enriched in lincRNA, snoRNA, and snRNA, whereas LD exRNA features mitochondrial rRNA, tRNA, and full-length ribosomal RNA. Proteomic and electron microscopy analyses link both fractions to extracellular vesicles, with HD proteins associated with nuclear components and LD proteins linked to mitochondria. These findings highlight the diversity of exRNA types and their complex biological roles (50). Several studies have also confirmed that EVs released by various cultured cells possess distinct RNA signatures, including sequences mapped to ncRNA including rRNA, Y-RNA, snRNA, tRNA and genomic repeats such as long interspersed nuclear elements (LINE), short interspersed nuclear elements (SINE), and long terminal repeats (LTR) elements (51–53).

Metabolomics provides insights into the small-molecule metabolites carried by EVs, which can affect metabolic pathways in recipient cells. The EV lipid signature discriminates ST-elevation myocardial infarction (STEMI) patients, and these findings may contribute to the identification of novel biomarkers and signaling mechanisms related to cardiac ischemia (54). In a detailed lipidomic study of exosomes from metastatic prostate cancer PC-3 cells, researchers identified approximately 280 lipid species, revealing significant differences in lipid composition between exosomes and their parent cells. Exosomes were notably enriched in glycosphingolipids, sphingomyelin, cholesterol, and phosphatidylserine, with an 8.4-fold higher lipid-to-protein ratio compared to parent cells, and exhibited selective lipid sorting that may inform mechanisms of exosome formation and function (55).

By integrating data from these different omics layers, researchers can gain a holistic understanding of the composition and function of EVs, particularly in the context of CVDs (Table 1). Moreover, the integration of these omic approaches has profound implications for EVs biology. In a study exploring proteome dynamics in a mouse model of pathological cardiac hypertrophy, the integration of transcript abundance, protein abundance, and protein turnover data led to a 75% increase in the identification of disease gene candidates. Importantly, protein turnover data revealed insights into post-transcriptional regulation and implicated unique disease-associated proteins that were not identified through transcript or protein abundance alone (66). Using proteomics and vesiculomics, Mark et al. identified disease-specific EV cargoes, including 773 proteins and 80 microRNAs, and linked them to procalcific Notch and Wnt signaling in carotid arteries and aortic valves, respectively (67). The combined analysis of proteomics and miRNA omics confirmed the distinctive profile of purified eFat-EVs from patients with atrial fibrillation (AF). *in vitro*, purified and unpurified eFat-EVs from patients with AF had a greater effect on proliferation and migration of human mesenchymal stromal cells and endothelial cells, compared with eFat-EVs from patients without AF (68). In summary, these techniques enable the identification of disease-specific EVs signatures, advancing the discovery of biomarkers for early diagnosis and prognosis.

**Table 1:** Multiomics analysis of EVs in cardiovascular disease.

Omics approach	Type of CVDs	Key molecules identified	Source of EVs	Biological role in CVDs	Ref
Proteomics	MI	C1QA, C5, APOD, APOC3, GP1BA, PPBP	Plasma	Postinfarct pathways of complement activation	(56)
Proteomics	MI	PLG, C8B, F2	Plasma	Tissue repair, cell proliferation, angiogenesis and maintaining vascular integrity	(57)
Proteomics	Hypertension	OLFM4, AT3, MPO	Urine	Glycosaminoglycan degradation, coagulation and complement system, and oxidative stress	(58)
Proteomics	MI-I/R	PAPP-A, NID1	CPC-EVs	IGF-R signaling pathway and angiogenesis.	(59)
Transcriptomics	MI	miR-4516 miR-203	Plasma	SFRP1 may be involved in lipid metabolism	(60)
Transcriptomics	MI	lncRNA-UCA1	hMSC	Promoting anti-apoptotic effects.	(61)
Transcriptomics	MI	lncRNA- HCG15	Serum	Facilitated cardiomyocyte apoptosis and inflammatory cytokine production.	(62)
Transcriptomics	CHD	S1PR5 CARNS1	Plasma	Myocardial fibrosis, endothelial cell permeability, and macrophage efferocytosis.	(63)
Metabolomics	Hypercholesterolemia	Phosphatidyl-cholines	CM-EVs	EVs secretion, lipid metabolism, adipocyte differentiation	(64)
Lipidomics	STEMI	Sphingomyelins	Plasma	Endothelial cell function, and inflammatory signaling	(54)
Lipidomics	MI	Phosphatidylcholine, sphingo-myelins, triglycerides	MSC-EVs	Tissue regeneration and regulation of the immune system	(65)

### 3.2 Key molecular insights from multiomics analyses of EVs in cardiovascular disease

Multiomics studies have provided critical insights into the role of EVs in CVDs by identifying key proteins, RNAs, and metabolites that contribute to disease progression and therapeutic responses. One of the significant findings from these studies is the identification of specific proteins within EVs that are associated with cardiovascular pathologies (69). For example, proteomic analyses have revealed that EVs derived from cardiovascular cells often contain proteins involved in inflammation, coagulation, and extracellular matrix remodeling (18). These proteins not only serve as biomarkers for the early detection of CVDs but also offer potential therapeutic targets. By modulating the levels or activities of these proteins within engineered EVs, researchers are exploring new ways to mitigate the adverse effects of CVDs, such as reducing inflammation or preventing reactive oxygen species overaccumulation. A study by Shen et al. presented a two-step EVs delivery system that enhances the cardiac targeting and therapeutic efficacy of curcumin for myocardial infarction while minimizing systemic toxicity and avoiding entrapment by the mononuclear phagocyte system (70). Furthermore, multiomics analyses have highlighted the presence of cardioprotective molecules or biomarkers within EVs, such as antiapoptotic factors or matrix metalloproteinases, which can be leveraged in therapeutic applications to increase the resilience of cardiac tissue to stress and injury (71, 72).

In addition to proteins, the RNA content of EVs has emerged as a crucial component in CVDs, particularly noncoding RNAs such as microRNAs (miRNAs) and long noncoding RNAs (lncRNAs). Multiomics studies have identified specific miRNAs within EVs that regulate key pathways involved in cardiac hypertrophy, fibrosis, and angiogenesis. For example, certain miRNAs in EVs have been shown to inhibit profibrotic signaling pathways, thereby reducing cardiac fibrosis and improving heart function following heart failure. For example, a study by Liu et al. revealed that decreased levels of exosomal miR-425 and miR-744 in plasma indicate the development of fibrosis during heart failure (73). Other miRNAs promote angiogenesis by targeting antiangiogenic factors, facilitating the repair of ischemic tissues. For example, cardiosphere-derived EVs enhanced heart function in a mouse model of MI by utilizing miRNA-146 to reduce apoptosis and inflammation while promoting cardiomyocyte proliferation and angiogenesis (74). The ability to manipulate the RNA cargo of EVs through engineering techniques opens new avenues for therapeutic interventions, where tailored EVs can deliver specific miRNAs to modulate disease processes in a controlled manner. Overall, the discovery of novel ncRNAs within EVs has expanded our understanding of the regulatory networks involved in CVDs, providing further opportunities for the development of RNA-based therapies.

Metabolomics, though less explored than proteomics and transcriptomics, has also contributed valuable insights into the role of EVs in CVDs. Metabolomic analyses of EVs have identified metabolites involved in energy metabolism, oxidative stress, and lipid signaling, all of which are critical in the context of cardiovascular health (75). Currently, therapeutic approaches that utilize EVs to deliver lipid metabolites for CVDs treatment are limited. The primary objective of metabolomics focused on EVs is to diagnose and identify potential lipid biomarkers associated with disease. For example, a study by Barile et al. demonstrated that the lipidomic signature of EVs, particularly sphingolipids, can accurately differentiate STEMI patients from controls, highlighting their potential as novel biomarkers for cardiac ischemia (54). These findings highlight the promising role of metabolomics in uncovering new therapeutic strategies, where EVs could be strategically engineered to deliver targeted metabolites, potentially restoring metabolic balance and offering protection against the progression of cardiovascular diseases.

## 4 Engineering extracellular vesicles for therapeutic applications in cardiovascular disease

### 4.1 Strategies for enhancing EVs cargo and targeting efficiency

The therapeutic potential of EVs in CVDs is significantly amplified through strategic engineering to increase their cargo and targeting efficiency (76). One approach involves the genetic modification of donor cells to overexpress specific therapeutic molecules, such as cardioprotective proteins or anti-inflammatory miRNAs, which are subsequently packaged into EVs (77–79). Additionally, physicochemical modifications can be employed to load EVs with small-molecule drugs, peptides, or nucleic acids. The study of Sun et al. has encapsulated curcumin into EVs through physical mixing, which not only retains the stability of curcumin *in vitro*, but also enhances the bioavailability of curcumin *in vivo* (80). Besides, a study used iron oxide nanoparticles (IONPs)-incorporated MSCs (IONP-MSCs) to develop exosome-mimetic EVs, which showed increased retention in infarcted hearts under magnetic guidance. The approach enhanced the levels of therapeutic molecules in IONP-MSCs and IONP-NVs, addressing challenges such as low exosome productivity (81).

To improve targeting efficiency, surface modification of EVs with specific ligands or antibodies that recognize receptors on diseased cardiovascular tissues is a promising strategy (82). Magnetic nanoparticles with an Fe3O4 core and a silica shell, functionalized with antibodies targeting CD63 on EVs and myosin-light-chain markers on injured cardiomyocytes, have demonstrated efficacy in myocardial infarction models by locally capturing and releasing EVs (83). These modifications can guide EVs to sites of injury or inflammation, thereby increasing the localized therapeutic effect while minimizing off-target effects. These engineered EVs offer a more precise and effective approach for treating CVDs, addressing the limitations of conventional therapies.

### 4.2 Targeted delivery and functional modulation of engineered EVs

Improving heart targeting while minimizing non-specific absorption and enhancing uptake efficiency by target cells are key challenges in this field (84). One of the most critical challenges is ensuring that these vesicles are delivered specifically to affected tissues, such as the myocardium or vascular endothelium, and that they retain their therapeutic function once they reach their target (85, 86). To achieve this goal, several innovative strategies have been developed to enhance the targeted delivery of engineered EVs. One approach involves the use of tissue-specific targeting ligands, such as peptides, antibodies, or aptamers, which are conjugated to the surface of EVs (87, 88). These ligands can recognize and bind to receptors that are overexpressed in diseased cardiovascular tissues, ensuring that the EVs are preferentially taken up by the target cells (89, 90).

For example, a study from Cheng et al. revealed that exosomes conjugated with a cardiac homing peptide (CHP) improve targeted delivery to the infarcted heart, enhancing cardiac function, reducing fibrosis, and promoting angiogenesis following myocardial infarction (88). Integrins on EVs surfaces facilitate targeted interactions with recipient cells through the recognition of extracellular matrix components or specific cell surface ligands. This integrin-mediated binding not only enables efficient internalization of EVs via endocytic pathways but also modulates downstream signaling in recipient cells. Studies have shown that blocking integrins *α*v/*β*3 on the cell surface significantly reduces the uptake of EVs (91, 92). Given their critical roles in myocardial fibrosis, integrins present promising targets for future therapeutic strategies, including EV-based approaches aimed at targeting the myocardium. By modulating integrin pathways, such therapies could reduce fibrosis and improve cardiovascular outcomes following heart injury (93).

Reducing off-target uptake by other tissues is essential to increase the therapeutic impact of EVs and reduce systemic side effects. A study introduced a two-step exosome delivery strategy to enhance targeting by first blocking macrophage uptake with “exosomeblocking” (siClathrin-loaded exosomes), followed by delivering “exosometherapeutic” (miR-21a-loaded exosomes). This method improved heart-specific delivery and significantly enhanced cardiac function in a doxorubicin-induced cardiotoxicity model, offering a promising approach for targeted gene therapy (94).

**Figure 2:**
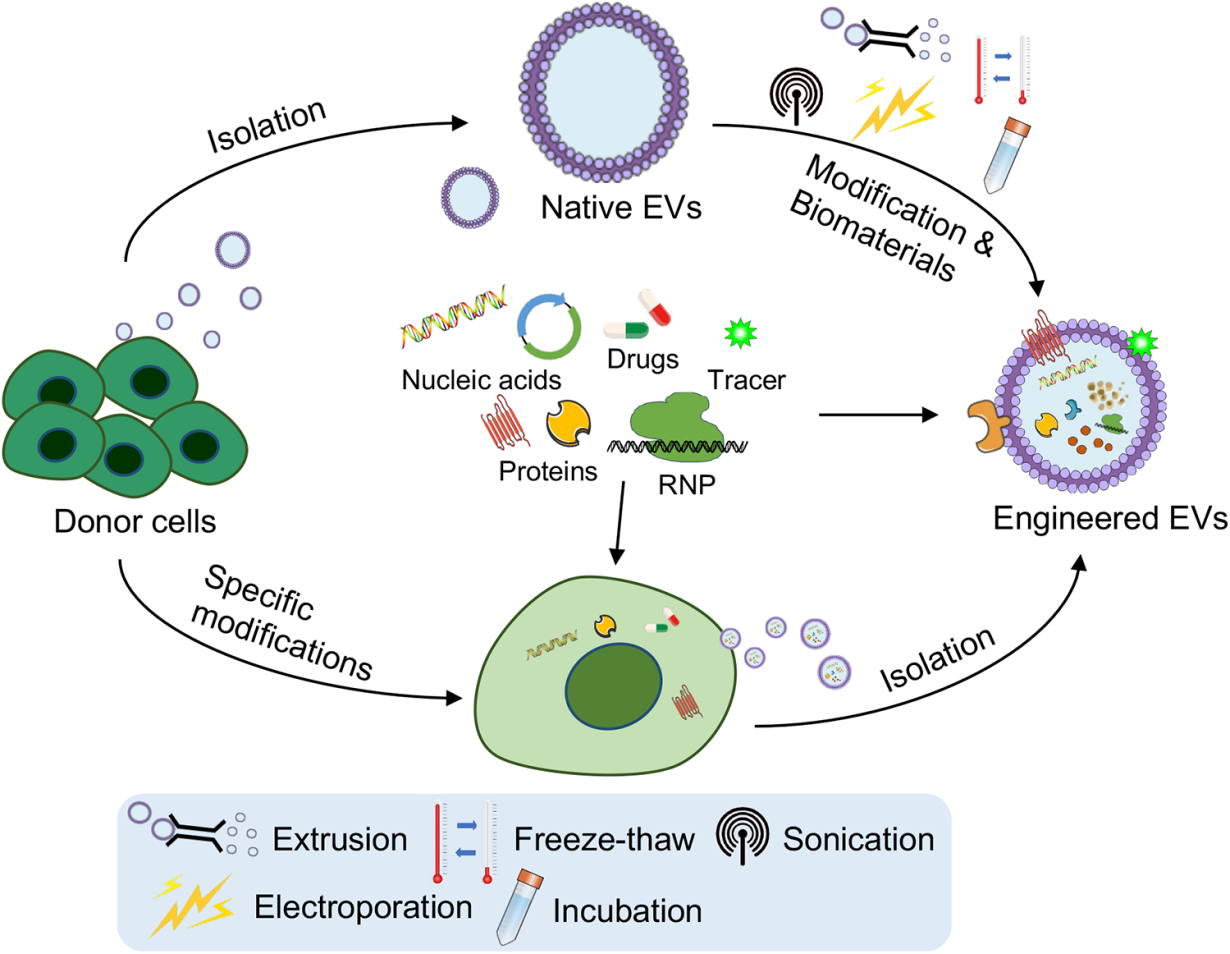
Engineering strategies for the targeting and functional modulation of EVs. Strategies such as genetic modification of parent (donor) cells to overexpress therapeutic nucleic acids or proteins, surface modification for improved targeting (e.g., cardiac-homing peptides), and cargo loading techniques (e.g., extrusion, freeze–thaw cycles, sonication, electroporation, incubation or combination with biomaterials) have been developed. These strategies aim to improve targeted delivery to cardiovascular tissues, functional modulation of EVs, and their ability to support angiogenesis, reduce fibrosis, and repair damaged heart tissue. The figure was partly generated via Servier Medical Art, provided by Servier, licensed under a CC BY 3.0.

In addition, the functional modulation of EVs plays a crucial role in optimizing their therapeutic efficacy. This can involve engineering EVs to carry specific signaling molecules that activate regenerative pathways or inhibit pathological processes within the cardiovascular system (76, 95). For example, EVs can be loaded with miRNAs or other types of RNAs that modulate gene expression in recipient cells, leading to the suppression of proinflammatory or profibrotic pathways (96–98). This modulation is particularly important in conditions such as heart failure or atherosclerosis, where the progression of the disease is driven by chronic inflammation and fibrosis (99, 100). By precisely modulating these pathways, engineered EVs can help restore normal tissue function and prevent further damage (Figure 2).

Furthermore, advances in bioengineering have allowed the development of engineered EVs that can respond to specific stimuli in the cardiovascular environment. These EVs can be designed to release their therapeutic cargo in response to changes in pH, proinﬂammatory cytokines, the adverse inﬁltration of neutrophils, the mobilization of monocytes and the degradation of the ECM (101). For example, a study presented an injectable conductive hydrogel (Gel@Exo) that binds exosomes from human umbilical cord mesenchymal stem cells, featuring conductivity matching the native myocardium, soft and dynamic stability adapting to heartbeats, and excellent cytocompatibility, significantly improving cardiac function and reducing fibrosis in a rat model of myocardial infarction-ischemia/reperfusion (MI-I/R) (102). This level of control not only enhances the effectiveness of EV-based therapies but also reduces the risk of side effects, making them safer options for the long-term treatment of CVDs (Figure 2).

### 4.3 Improving the stability and functional longevity of engineered EVs

The clinical success of engineered EVs in treating CVDs also depends on their stability and functional longevity in the circulatory system. Although they are inherently stable, EVs can be further optimized to resist degradation and maintain their therapeutic function over extended periods. Strategies to increase EVs stability include surface modifications with polyethylene glycol (PEG) to prevent rapid clearance by the immune system and the encapsulation of EVs in protective hydrogels or nanomaterials that prolong their circulation time (103, 104). Additionally, engineering EVs to carry antioxidant enzymes or molecules holds great promise for protecting their cargo from oxidative stress, offering a hopeful avenue for combating CVDs more effectively in the future (80, 105). By ensuring that EVs remain functional and stable until they reach their target tissue, these strategies significantly improve the potential of EV-based therapies to provide sustained therapeutic effects in CVDs management.

## 5 Potential and challenges of EV-based therapies for cardiovascular disease

### 5.1 Therapeutic potential of EVs in cardiovascular disease

EVs hold immense promise as therapeutic agents in the treatment of CVDs, offering a novel approach that leverages their natural roles in intercellular communication and tissue repair. EVs, particularly those derived from stem cells, have been shown to promote tissue regeneration, reduce inflammation, and improve cardiac function following myocardial infarction or other cardiac injuries. Their ability to deliver diverse cargoes of proteins, lipids, and nucleic acids directly to target cells makes them uniquely suited for addressing the complex pathology of CVDs. By modulating key signaling pathways and promoting cellular repair processes, EVs could revolutionize the way CVDs is treated, moving beyond traditional pharmacological interventions to more targeted, regenerative therapies.

### 5.2 Challenges in the development and clinical translation of EV-based therapies

Despite the promising therapeutic potential of EVs, significant challenges must be addressed to facilitate the transition from experimental research to clinical application. One of the primary challenges is the large-scale production and standardization of EVs (106). The process of isolating and purifying EVs is complex and labor intensive and requires sophisticated techniques such as ultracentrifugation, size-exclusion chromatography, or immunoaffinity capture. Variability in the production process can lead to inconsistencies in the quality and potency of EVs preparations, posing a significant hurdle for their use in clinical settings. Furthermore, the lack of standardized protocols for EVs isolation, characterization, and storage complicates the comparison of results across different studies and hinders the establishment of reliable therapeutic products (107). Addressing these issues will require the development of more efficient and scalable production methods, as well as the establishment of rigorous standards and quality control measures for EV-based therapies.

Advances in bioreactor systems and microfluidic technologies have increased yields and streamlined purification while maintaining bioactivity (108). Current frameworks, such as the minimal information for studies of extracellular vesicles (MISEV) guidelines, require refinement to meet clinical needs, including harmonization of parameters such as particle size, purity, and bioactivity (109). Efforts by organizations such as the International Society for Extracellular Vesicles (ISEV) aim to establish robust quality control standards, validated potency assays, and critical quality attributes (CQAs) essential for regulatory compliance and reproducibility (110–112). Furthermore, regulatory challenges for EV-based therapies arise from ambiguous classification and varying criteria across agencies such as the Food and Drug Administration (FDA) and the European Medicines Agency (EMA). Addressing these issues requires early engagement with regulators and international collaboration to harmonize manufacturing standards and product characterization.

**Figure 3:**
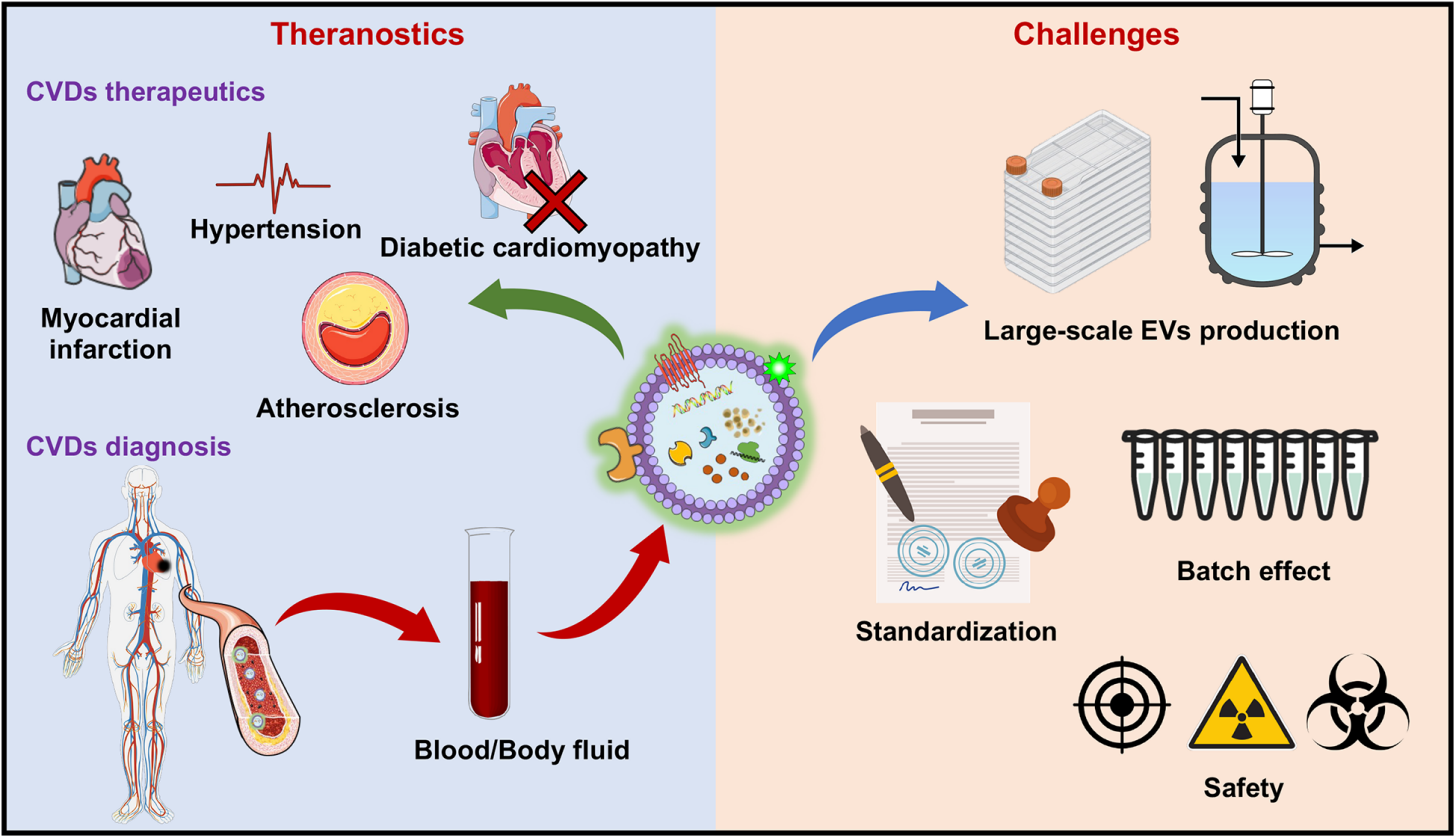
Therapeutic applications and challenges of EV-based therapies for CVDs. The therapeutic application**s** of EVs include various CVDs, including myocardial infarction (MI), hypertension, atherosclerosis, and diabetic cardiomyopathy, where EVs promote tissue repair, reduce inflammation, and increase angiogenesis. On the diagnostic side, EVs show promise as biomarkers for CVDs, with their lipid, protein, and RNA contents in blood and body fluids providing critical information for early detection and disease monitoring. The challenges faced in the clinical translation of EV-based therapies include issues related to large-scale production, standardization of isolation and characterization techniques, the “batch effect” leading to variability in EVs properties, and concerns over the safety of EV-based treatments (e.g., off-target effects, immunogenicity of EVs and radioactivity of the tracer). These barriers must be overcome to fully realize the potential of EVs in CVDs theranostics. The figure was partly generated via Servier Medical Art, provided by Servier, licensed under a CC BY 3.0.

Another critical challenge is the safety and regulatory approval of EV-based therapies. While EVs are generally considered to have low immunogenicity and good biocompatibility, there are still concerns regarding the heterogeneity of EVs populations, along with the complexity of their cargo, making it difficult to predict and control their biological activity (113). Evaluating the long-term safety of EV-based therapies is critical to their clinical success and widespread adoption. Key safety concerns include tumorigenicity, immune modulation, off-target effects, and unexpected toxicities (114). Current clinical trials often lack extended follow-up periods, underscoring the need for comprehensive long-term monitoring protocols (115). Moreover, advanced imaging technologies and biomarker-based approaches offer innovative solutions for real-time safety assessment, enabling a deeper understanding of biodistribution, accumulation, and clearance over time (116). Regulatory agencies need to establish clear guidelines for the safety assessment of EV-based products, including rigorous preclinical testing and well-designed clinical trials, to evaluate their efficacy and potential risks (117). Additionally, establishing centralized databases to aggregate trial data across studies is vital for identifying rare adverse events, conducting meta-analyses, and setting safety benchmarks (118) (Figure 3). These efforts will provide essential insights into the long-term viability and regulatory acceptance of EV-based therapies.

### 5.3 Future directions for EV-based cardiovascular therapies

To fully realize the potential of EV-based therapies for CVDs, future research must focus on overcoming current limitations and advancing the field through innovative approaches. One key area is improving the precision of engineering techniques to increase both the targeting and functional capabilities of EVs (113, 119). Advances in bioengineering, such as surface modification and genetic manipulation, are essential for creating EVs that can deliver therapeutic molecules with greater specificity to diseased cardiovascular tissues (120). Additionally, improving the scalability of EVs production is critical to meet clinical demand. Techniques such as microfluidic devices for EVs isolation or the use of bioreactors to culture donor cells may help produce EVs at a larger scale without compromising their quality (121, 122). Moreover, enhancing the stability of EVs in circulation, perhaps by optimizing their lipid composition or through encapsulation strategies, will be essential for increasing their longevity and therapeutic efficacy in patients with CVDs in the near future (123–125).

The integration of EV-based therapies with other emerging technologies holds significant promise for the development of next-generation therapeutics. For example, CRISPR/Cas9-based technologies have revolutionized gene editing, and their application in EVs offers tremendous potential for targeted therapeutic strategies. Combining EV-based delivery systems with gene editing technologies, such as CRISPR/Cas9, could allow for highly targeted interventions at the genetic level, correcting the molecular defects that contribute to cardiovascular disease (125, 126). By editing the cargo of EVs, such as RNA or proteins, we can design EVs with enhanced therapeutic properties or more specific targeting capabilities. A study developed CRISPR-Cas9-loaded EVs with cardiac-targeting peptides for precise genome editing of miR-34a in myocardial infarction. This system reduces apoptosis, improves cardiac function, and shows promise as a tissue-specific gene therapy for cardiovascular disease (127). This strategy could address some of the limitations associated with the current heterogeneity of EVs and improve their consistency and efficacy in clinical applications.

Synthetic biology approaches also offer the possibility of designing EVs with customized properties tailored to specific therapeutic needs, such as engineered EVs that respond to environmental stimuli within the cardiovascular system (88, 128). Therefore, artificial or synthetic EVs offer a promising alternative to natural EVs by enabling controlled and scalable production processes. A previous study demonstrated that artificial leukosomes, which are biomimetic nanovesicles that combine liposome and leukocyte properties, are effective vehicles for targeted doxorubicin delivery and significantly inhibit tumor growth in breast cancer and melanoma models (129). Chen et al. developed cationic biomimetic EVs via a microemulsion and micelle assembly method, incorporating DEC205 monoclonal antibodies for dendritic cell (DC) targeting together with reduced cytotoxicity and enhanced cellular uptake, highlighting their potential as antigen carriers for specific DC targeting (130). These engineered vesicles can be designed to mimic the structure and function of natural EVs while addressing challenges such as low yield and batch variability. Additionally, their cell-free nature reduces the risks associated with donor variability or immune responses. Collaborations among researchers, clinicians, and regulatory bodies will be critical to ensure the safe and effective translation of these advanced EV-based therapies into clinical practice (131–133). Continued preclinical research, rigorous clinical trials, and regulatory frameworks will be needed to ensure that these innovative approaches can provide safe, effective, and accessible treatment options for patients with cardiovascular disease.

## 6 Conclusion

This review highlights the significant role of EVs in CVDs treatment. EVs, particularly exosomes, facilitate intercellular communication by delivering essential molecular cargo, including proteins, lipids, and nucleic acids, to modulate disease progression and tissue repair. Through multiomics analyses, we identified key molecular players within EVs that can serve as both biomarkers and therapeutic agents in CVDs. Furthermore, the engineering of EVs for enhanced cargo delivery and targeting has shown promise in the development of more effective and precise therapeutic strategies. Although challenges such as production scalability and standardization remain, the potential of EVs in cardiovascular therapy is undeniable.

The future of EV-based therapies holds great promise for more personalized and targeted treatment approaches in CVDs. As engineering techniques advance, EVs can be designed to deliver therapeutic molecules tailored to individual patients, moving beyond traditional treatments. The integration of EVs with technologies such as gene editing and synthetic biology could further increase their therapeutic potential, offering precise interventions that address the underlying molecular causes of CVDs. With continued innovation, EV-based treatments could dramatically change the landscape of CVDs therapy, providing more effective solutions for patients.
